# Sea Cucumber Derived Triterpenoid Glycoside Frondoside A: A Potential Anti-Bladder Cancer Drug

**DOI:** 10.3390/nu15020378

**Published:** 2023-01-11

**Authors:** Ruizhen Ru, Gengzhan Chen, Xiaoxia Liang, Xudong Cao, Lihong Yuan, Minjie Meng

**Affiliations:** 1 Guangdong Province Key Laboratory for Biotechnology Drug Candidates, School of Biosciences and Biopharmaceutics, Guangdong Pharmaceutical University, Guangzhou 510006, China; 2 Department of Chemical and Biological Engineering, University of Ottawa, Ottawa, ON K1N6N5, Canada

**Keywords:** bladder cancer, Frondoside A, CpG oligodexynucleotides, cell viability, apoptosis, cell migration

## Abstract

Bladder cancer is a highly recurrent disease and a common cause of cancer-related deaths worldwide. Despite recent developments in diagnosis and therapy, the clinical outcome of bladder cancer remains poor; therefore, novel anti-bladder cancer drugs are urgently needed. Natural bioactive substances extracted from marine organisms such as sea cucumbers, scallops, and sea urchins are believed to have anti-cancer activity with high effectiveness and less toxicity. Frondoside A is a triterpenoid glycoside isolated from sea cucumber, *Cucumaria frondosa*. It has been demonstrated that Frondoside A exhibits anti-proliferative, anti-invasive, anti-angiogenic, anti-cancer, and potent immunomodulatory effects. In addition, CpG oligodeoxynucleotide (CpG-ODN) has also been shown to have potent anti-cancer effects in various tumors models, such as liver cancer, breast cancer, and bladder cancer. However, very few studies have investigated the effectiveness of Frondoside A against bladder cancer alone or in combination with CpG-ODN. In this study, we first investigated the individual effects of both Frondoside A and CpG-ODN and subsequently studied their combined effects on human bladder cancer cell viability, migration, apoptosis, and cell cycle in vitro, and on tumor growth in nude mice using human bladder cancer cell line UM-UC-3. To interrogate possible synergistic effects, combinations of different concentrations of the two drugs were used. Our data showed that Frondoside A decreased the viability of bladder cancer cells UM-UC-3 in a concentration-dependent manner, and its inhibitory effect on cell viability (2.5 μM) was superior to EPI (10 μM). We also showed that Frondoside A inhibited UM-UC-3 cell migration, affected the distribution of cell cycle and induced cell apoptosis in concentration-dependent manners, which effectively increased the sub-G1 (apoptotic) cell fraction. In addition, we also demonstrated that immunomodulator CpG-ODN could synergistically potentiate the inhibitory effects of Frondoside A on the proliferation and migration of human bladder cancer cell line UM-UC-3. In in vivo experiments, Frondoside A (800 μg/kg/day i.p. for 14 days) alone and in combination with CpG-ODN (1 mg/kg/dose i.p.) significantly decreased the growth of UM-UC-3 tumor xenografts, without any significant toxic side-effects; however, the chemotherapeutic agent EPI caused weight loss in nude mice. Taken together, these findings indicated that Frondoside A in combination with CpG-ODN is a promising therapeutic strategy for bladder cancer.

## 1. Introduction

Bladder cancer is a commonly diagnosed cancer (3.0% of total cancer cases) and is one of the leading causes of cancer death (2.1% of total cancer deaths). The incidence and mortality rates of bladder cancer have steadily increased, and it has been estimated that there were 573,278 new cases and 212,536 deaths worldwide in 2020 [1]. There are two main types of bladder cancer: muscle-invasive bladder cancer (MIBC) and non-muscle-invasive bladder cancer (NMIBC), and approximately 75% of bladder cancers are NMIBC [2]. To treat bladder cancers, several chemotherapy drugs, such as Epirubicin, Gemcitabine, and Cisplatin are currently in use [3]. For the treatment of NMIBC, Bacillus Calmette-Guérin (BCG) instillation is considered as the gold standard; however, the BCG treatment is not effective against the NMIBC in about 40% of the patients [2], and its clinical application is limited by high recurrence rate, poor prognosis, low five years overall survival rates, postoperative complications, and poor tolerance of treatments [2]. As a result, there is a pressing need to find a novel anti-bladder cancer drug from natural sources to develop effective therapies for bladder cancer treatment.

Natural source drugs, which are derived or extracted from plants, microbes, and marine organisms, currently account for more than 60% of anti-cancer drugs [4,5]. Marine organisms have proven to be an exceptionally valuable resource for bio-active natural products, particularly in the area of potential anti-cancer compounds. Sea cucumbers, a marine invertebrate, have been used in traditional medicine in Asia for centuries [6]. Sea cucumbers contain numerous bioactive compounds such as saponins, peptides, and glycosaminoglycan; they have been shown to have many interesting pharmacological effects, such as anti-oxidant, anti-bacterial, and anti-cancer effects, and contribute to preventing chronic diseases [7,8]. Frondoside A (see Appendix A for chemical structure) is a triterpenoid glycoside isolated and purified from sea cucumber *Cucumaria frondosa* [9,10]; it has been known to have anti-proliferative [11], anti-invasive [12], anti-angiogenic [12], anti-cancer [13], and immunomodulatory effects [14,15]. For example, Frondoside A has been shown to have anti-cancer effects on cancers, such as pancreatic cancer [16,17], breast cancer [18,19], lung cancer [12], prostate cancer [20], and leukemia [21,22]. However, to date, there are very few studies evaluating the use of Frondoside A-based treatment for bladder cancers.

CpG oligodeoxynucleotide (CpG-ODN) is a synthetic unmethylated G-rich and C-rich sequence. As a Toll-like receptor-9 (TLR9) agonist, CpG-ODN is known to induce anti-cancer immune responses and exert direct effects against cancer cells, serving as a cancer therapeutic agent [23,24]. The TLR9 can be activated by bacterial and viral infections, immunoglobuline-DNA complexes, and synthetic ODNs, which contain unmethylated CpG sequences [25]; it can also be activated by its ligands, such as CpG-ODN. Therefore, TLR9 can recognize foreign unmethylated CpG DNA, and activate a strong Th 1 response that plays a critical role in the innate and adaptive immune responses [26]. In our previous studies, we demonstrated that CpG-ODN could be a promising treatment option for various cancers including lung, liver [27], and bladder cancers [28,29]; in addition, we showed that CpG-ODN could also be used as a potential vaccine candidate to develop an effective vaccine against human cancers [30,31].

In the current study, we investigated the individual anti-bladder cancer effects of both Frondoside A and CpG-ODN and those of the combination of Frondoside A and CpG-ODN in vitro and in vivo. To our knowledge, this is the first experimental investigation to study the combined effects of triterpenoid glycoside Frondoside A (extracted from sea cucumber *Cucumaria frondosa*) and CpG-ODN against human bladder cancer.

## 2. Materials and Methods

### 2.1. Animal and Ethics Statement

BALB/c nude mice (nu/nu) were purchased from Guangdong Medical Laboratory Animal Center. All animal experiments were established using the ARRIVE guidelines and carried out in accordance with the United Kingdom Animals (Scientific Procedures) Act of 1986 and associated guidelines, the European Union Directive 2010/63/EU for animal experiments. The animal experiments were performed in accordance with the protocol approved by the Animal Ethics Committee and the Institutional Animal Care at Guangdong Pharmaceutical University (identification No. SPF2020004).

### 2.2. Reagents

Triterpene glycoside Frondoside A (extracted from *Cucumaria frondosa,* purity > 98% determined by HPLC) was purchased from Kerafast (Boston, MA, USA). Cell Counting kit-8 (CCK-8) was purchased from US EVERBRIGHT^®^ Inc. (Suzhou, Jiangsu, China). Hoechst-33258 Staining Kit, Annexin-V-FITC Apoptosis Detection Kit, and Cell Cycle and Apoptosis Analysis Kit were obtained from Beyotime (Zhenjiang, Jiangsu, China). α-MEM medium, fetal bovine serum (FBS), PBS, penicillin (100 Units/mL), streptomycin (100 μg/mL), 0.25% Trypsin (1X), and Nonessential Amino Acid Solution (NAAS, 100X) were purchased from Thermo Fisher Scientific (Waltham, MA, USA). PrimeScriptTMRT reagent Kit with gDNA Eraser (Perfect Real Time), TB Green^®^ Premix Ex TaqTM II (Tli RNaseH Plus), and RNase Free dH_2_O were obtained from TaKaRa Bio (TaKaRa, Dalian, China). Chemotherapetic agent Epirubicin hydrochloride (EPI), a commonly used chemotherapy drugs to treat bladder cancer in the clinic, was purchased from Aladdin (Shanghai, China).

### 2.3. CpG Oligodeoxynucleotide (CpG-ODN) Sequence

The CpG-ODN sequence (5′-AACGTTGTCGTCGACGTCGTCGTC-3′) was designed based on our previous study [29], and custom synthetized by Sangon (Shanghai, China) with phosphorothioate (PS) backbone.

### 2.4. Cell Lines and Cell Cultures

The human bladder cancer cell line UM-UC-3 (ATCC^®^ no. CRL-1749) was purchased from American Type Culture Collection (ATCC, Manassas, VA, USA). The UM-UC-3 cells were maintained in α-MEM medium supplemented with 10% FBS, 1% Nonessential Amino Acid Solution (NAAS), 100 units/mL of penicillin, and 100 μg/mL of streptomycin, in a humidified cell culture incubator at 37 °C with 5% CO_2_.

### 2.5. Cell Viability Assays

Cytotoxicity of single compounds or drug combinations was evaluated using the CCK-8 assay. In brief, UM-UC-3 cells were seeded into the wells of 96-well plates at a density of 5000 cells per well, incubated overnight, and treated with different concentrations of Frondoside A (0.01–2.5 μM) for pre-determined time durations (i.e., 24, 48, and 72 h). After the treatments, the UM-UC-3 cells were incubated in the CCK-8 reagent for 3 h and the cell viability was determined according to the manufacturer’s instructions. Optical density (OD) was measured at 450 nm using a Bio-Rad microplate reader (Hercules, CA, USA). In the second set of the experiments, cells were also treated for 24, 48, and 72 h with combinations of Frondoside A (0.25–0.75 μM) and CpG-ODN (0.001–1 μM), and the cell viability was assessed using the same procedure as discussed above. In parallel, EPI (10 μM) and PBS (pH 7.4) were also used as positive and blank controls, respectively. 

### 2.6. Examination of Synergistic/Antagonistic Effect of Drug Combination

Determination of either synergistic, antagonistic, or additive effects of the compounds when used in combination was performed using the Chou–Talalay method [32,33] based on the date collected in the CCK-8 assays. The combinational index (CI) was calculated with CompuSyn v.1.0 software (ComboSyn, Inc., Paramus, NJ, USA). Synergism is defined as a CI value is less than 0.85, whereas antagonism is defined by a CI value greater than 1.2. A CI value between 0.85 and 1.2 reflects an additive effect. 

### 2.7. Cell Migration Assay

The UM-UC-3 cells were cultured to confluence in 6-well plates, and carefully scratched with a plastic pipette tip. Subsequently, the plates were washed twice with PBS (pH 7.4) and incubated at 37 °C in fresh α-MEM containing 1% FBS in pre-determined concentrations of either Frondoside A alone (0.35–1 μM) or Frondoside A (0.5 μM) in combination with CpG-ODN (0.001–1 μM). In parallel, EPI (10 μM) was used as a positive control. The wound-healing area was continuously monitored at different time points (i.e., 12 h and 24 h) using an Olympus inverted microscope (Olympus, Tokyo, Japan). 

### 2.8. Cell Cycle Analysis

The cell cycle distribution was analyzed by flow cytometry using PI staining, according to the manufacturer’s instructions. More specifically, the UM-UC-3 cells was pre-incubated overnight in 6-well plates (2 × 10^5^ cells per well) and were subsequently treated with different concentrations of either Frondoside A alone or Frondoside A (0.5 μM) in combination with CpG-ODN (0.001–1 μM) for 48 h. Subsequently, cells were trypsinized, centrifuged, fixed with 75% ethanol (*v/v*), washed twice with PBS (pH 7.4), re-suspended in PBS (pH 7.4) containing PI and RNase A at 37 °C for 30 min, and finally quantitatively analyzed using a BD FACS Calibur Flow Cytometer (Franklin Lakes, NJ, USA). Data acquisition was performed using BD Bioscience Cell Quest Pro software (BD Bioscience, Franklin Lakes, NJ, USA). Fractions of cells in G0/G1, S and G2/M phases were determined using a FlowJo software (Version 10.0.7, Tree Star, Inc., Ashland, OR, USA).

### 2.9. Cell Apoptosis and Cell Morphology Analysis

Induction of apoptosis was examined by a FACS-based analysis with an Annexin-V-FITC and propidium iodide (PI) double staining. Specifically, UM-UC-3 cells was seeded in 6-well plates (2 × 10^5^ cells per well) and incubated overnight. The different concentrations of either Frondoside A alone (0.35–1 μM) or Frondoside A (0.5 μM) in combination with CpG-ODN (0.001–1 μM) were used to treat the cells for 48 h; in parallel, EPI (10 μM) was used as a positive control. After the treatments, the cells were harvested, re-suspended in 500 μL Annexin V-FITC binding buffer, stained with 5 μL Annexin V-FITC and 10 μL PI in the dark for 20 min, and analyzed using a BD Bioscience FACS Calibur flow cytometer. Induced apoptosis in the UM-UC-3 cells was assessed by Hoechst-33258 staining to observe the morphological changes of the nuclei [34]. The UM-UC-3 cells were seeded in 6-well plates at a density of 8 × 10^4^ cells per well overnight and the cells were treated with different concentrations of either Frondoside A alone or the combinations of Frondoside A and CpG-ODN for 48 h, as discussed above. Finally, the cells were stained by Hoechst-33258 for 5 min and observed under a fluorescence microscope (Olympus, Tokyo, Japan).

### 2.10. RNA Isolation and Quantitative Real-Time Polymerase Chain Reaction Analysis

UM-UC-3 cells were seeded in 6-well plates at a density of 4 × 10^5^ cells per well overnight. Subsequently, the cells were treated with either Frondoside A (0.5 μM), CpG-ODN (1 μM) individually, or in combination for 48 h; EPI (10 μM) was used as a positive control. After the treatments, total RNA was extracted from the cells using a Trizol Reagent (Life Technologies, Carlsbad, CA, USA). Real-time qPCR (Bio-Rad Laboratories, Hercules, CA, USA) was performed using SYBR Green qPCR master mix (TaKaRa, Dalian, China) according to the manufacturer’s protocol. Gene-specific primers used are listed in Table 1. Samples were normalized to the housekeeping gene, GAPDH. The relative RNA levels were determined with the 2^−∆∆Ct^ method.

### 2.11. In Vivo Bladder Cancer Xenograft Assay

For cancer xenografts, 4–6 weeks old male BALB/c nude mice (nu/nu) were obtained from the Guangdong Medical Laboratory Animal Center and bred in the animal facility. The mice were housed in micro-isolator cages in a filtered-aired laminar flow cabinet and handled under aseptic conditions. 

UM-UC-3 cells were used to inject into the mice. In brief, 100 μL of the cell suspension (a total of 3 million cells) were injected subcutaneously into the right flank of the mice. When tumors had reached the volume of 70–100 mm^3^, animals were randomly assigned to either a treatment or a control arm (5 mice per group). The animals were treated for 14 days with either Frondoside A (800 μg/kg/day, i.p.), CpG-ODN (1 mg/kg/dose, i.p.), or combination of Frondoside A (800 μg/kg/day, i.p.) and CpG-ODN (1 mg/kg/dose, i.p.) treatment. Control animals were treated with either vehicle alone (0.9% saline) or EPI (1 mg/kg/dose, i.p.). Tumor dimensions and animal weights were monitored every 2 days. Because of animal ethical requirements, animals in all groups were sacrificed when tumor sizes of blank control group were greater than a pre-set endpoint volume of 1000 mm^3^. Once the animals were sacrificed, tumors and the main organs (heart, kidney, liver, spleen, and brain) of the animals were excised and weighed. Finally, the organ coefficient (a common toxicology indicator of ratio of organ and body weight reflecting animal damage) were calculated and the tumor tissues were fixed with 4% paraformaldehyde overnight and H&E stained.

### 2.12. Statistical Analysis

All results were presented as mean ± S.E.M of at least three repeats and plotted using GraphPad Prism software (Version 8.3.0). Statistical analysis was done using ANOVA; differences with *p* < 0.05 were considered statistically significant.

## 3. Results

### 3.1. Frondoside A Inhibits Bladder Cancer Cell Viability and Migration

**Figure 1:**
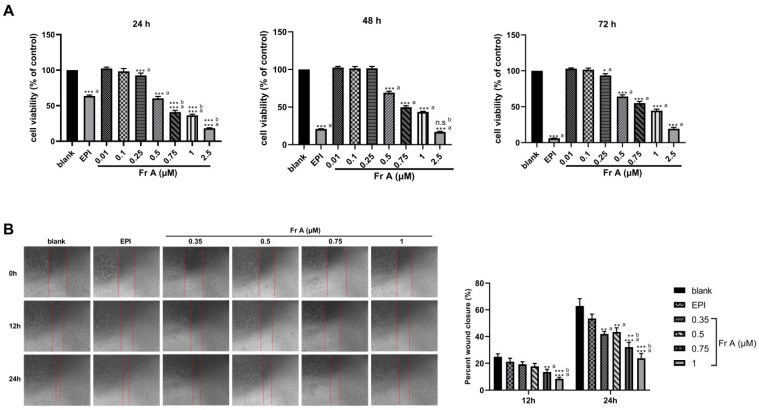
Inhibitions of cell viability and migration by Frondoside A (Fr A). Exponentially growing UM-UC-3 cells were treated with the indicated concentrations of Frondoside A for 24, 48, and 72 h (**A**). Cell viability was assayed as described in Materials and Methods. Frondoside A significantly suppressed the migration of UM-UC-3 cells, and cell mobility was measured at 0, 12, and 24 h (objective × 4) (**B**). Data are expressed as means ± S.E.M. * Significantly different at *p* < 0.05. ** Significantly different at *p* < 0.01. *** Significantly different at *p* < 0.001. n.s (not significant). “a” stands for versus blank control; “b” stands for versus EPI (10 μM).

As shown in Figure 1, Frondoside A reduced cell viability and migration in a concentration-dependent manner (Figure 1A,B). With the increasing concentrations of Frondoside A (0.01–2.5 μM), the cell viability was significantly decreased (*p* < 0.05). The IC_50_ of Frondoside A was determined to be approximately 0.75 μM for the UM-UC-3 cells (IC_50_ = 0.75 μM). When the cells were treated with Frondoside A at concentrations higher than 0.75 μM for 24 h, the inhibition of cell viability was superior to EPI (*p* < 0.001) (Figure 1A). In comparison, when the cells were treated with 2.5 μM Frondoside A for 48 h, the degree of inhibition was similar to cells treated with 10 μM EPI for 48 h (Figure 1A).

It is evident from Figure 1B that with increasing concentrations of Frondoside A (0.35–1 μM), the inhibitory effects on cell migration showed an increasing trend. It should also be noted that at concentration higher than 0.35 μM for both 12 and 24 h treatments, Frondoside A showed more significant inhibitory effects on migration than EPI treatment at a much higher concentration of 10 μM. More specifically, the migration rates of Frondoside A (0.35–1 μM) were 19.31% ± 1.88% to 8.58% ± 1.03% in 12 h, and 43.40% ± 3.13% to 23.77% ± 3.51 in 24 h, while the rates of EPI were 21.15% ± 2.71% and 53.51% ± 3.24% in 12 h and 24 h, respectively (Figure 1B).

### 3.2. Frondoside A Affects Bladder Cancer Cell Cycle Distribution

**Figure 2:**
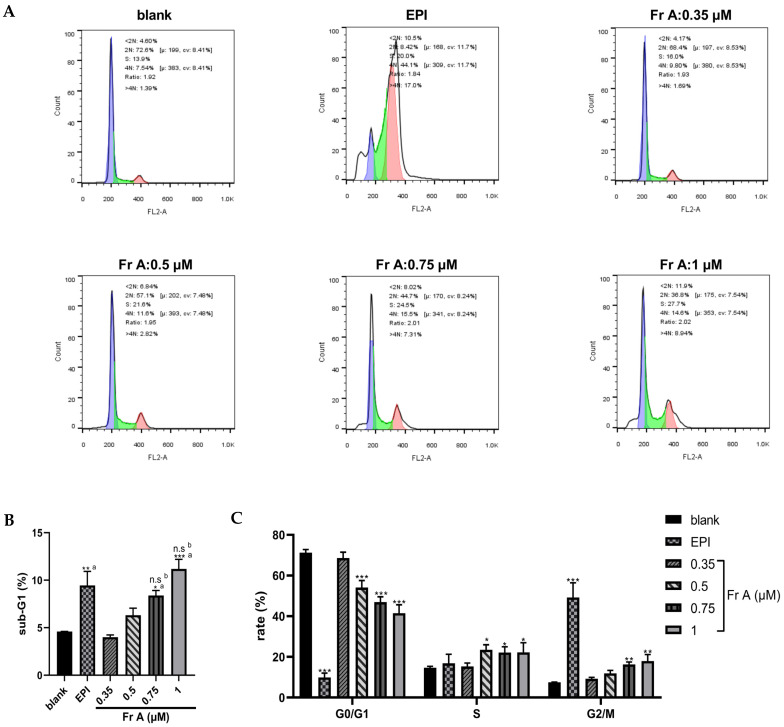
Effects of Frondoside A (Fr A) on cell cycle in bladder cancer cells. Cells were treated with increasing concentrations of Frondoside A for 48 h, with EPI (10 μM) as positive control. The distribution of cell cycle (**A**–**C**). Data are expressed as mean ± S.E.M. * Significantly different at *p* < 0.05. ** Significantly different at *p* < 0.01. *** Significantly different at *p* < 0.001. n.s (not significant). “a” stands for versus blank control; “b” stands for versus EPI (10 μM).

As shown in Figure 2, treatment with Frondoside A affected cell cycle distribution in UM-UC-3 bladder cancer cells. Treatments with increasing concentrations (0.35–1 μM) of Frondoside A significantly reduced the fraction of cells in the G0/G1 phase of the cell cycle while an evident increase in the fraction of cells in the S and G2/M phases (see Figure 2A,C) was noted. Additionally, Frondoside A (0.5–1 μM) treatment for 48 h induced a sub-G1 phase arrest, demonstrating that apoptosis is a major contributor to the response seen in this study, a similar effect also seen in cells treated with EPI at 10 μM. Furthermore, UM-UC-3 cells treated with Frondoside A (0.75–1 μM) for 48 h showed increased proportions of cells in sub-G1 phase that was similar or superior to those treated with EPI (10 μM) (Figure 2B).

### 3.3. Frondoside A Induces Cell Apoptosis and Change Nuclei Morphology

**Figure 3:**
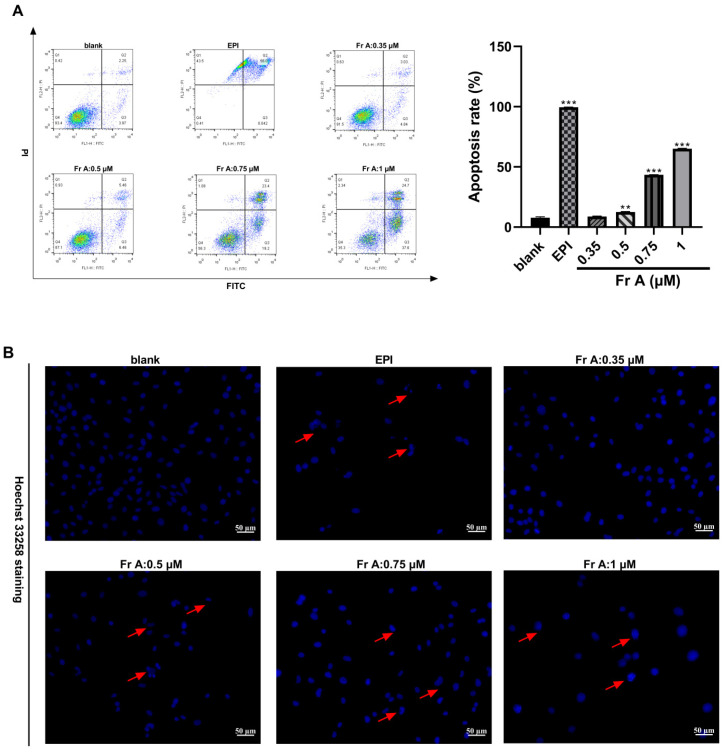
Effects of Frondoside A (Fr A) treatment on apoptosis and cell morphology in UM-UC-3 cells. Cells were treated with increasing concentrations of Frondoside A for 48 h, and EPI (10 μM) as positive control. The induction of cell apoptosis (**A**) and the morphological changes (the pictures were taken at ×200 magnification) (**B**). Data are expressed as mean ± S.E.M. ** Significantly different at *p* < 0.01. *** Significantly different at *p* < 0.001.

As shown in Figure 3A, Frondoside A treatment induced cell apoptosis in a concentration-dependent manner while EPI treatment (10 μM) induced cell death through both apoptosis and necrosis. For example, exposure to Frondoside A treatment at 0.5 μM, 0.75 μM, and 1 μM induced 10%, 40%, and 60% of the cells to undergo apoptosis, respectively. Similarly, a concentration-dependent morphological changes were also detected in Frondoside A treated UM-UC-3 cells, showing nuclear fragmentation, chromatin condensation, blebbing of the plasma membrane, and formation of apoptotic bodies (Figure 3B) under the phase contrast microscope. These morphological changes associated with apoptosis were evidently different from those in EPI treatment at 10 μM (Figure 3B).

### 3.4. CpG-ODN Enhances the Inhibition of Frondoside A on UM-UC-3 in Cell Viability and Migration Assays, but Has No Significant Altering on Cell Cycle Distribution, Apoptosis and Nuclei Morphology

**Figure 4:**
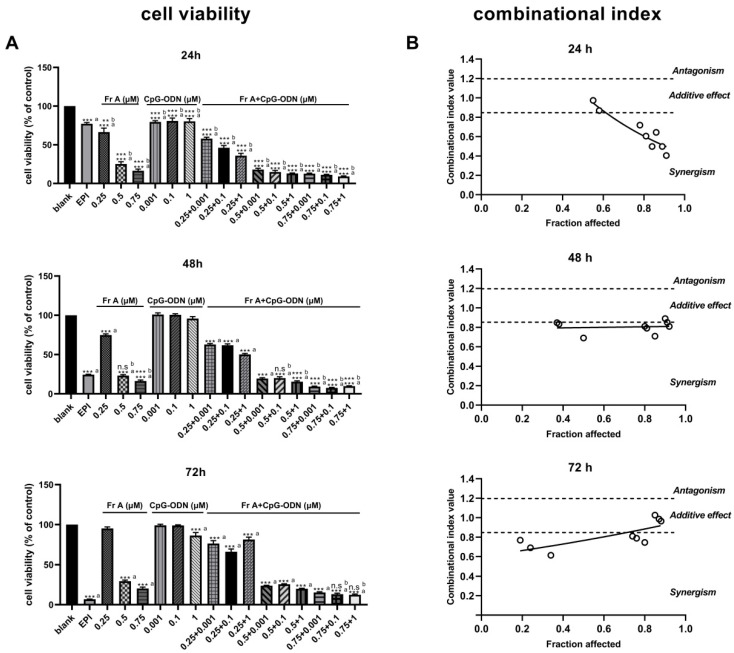

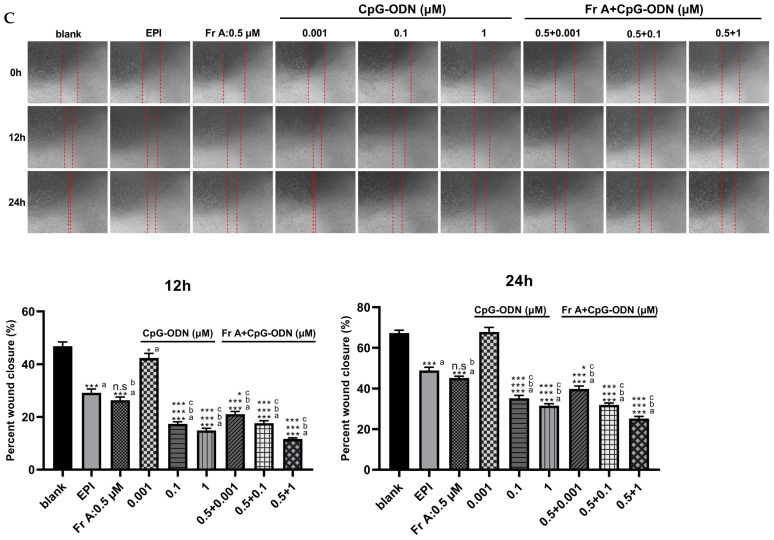
CpG-ODN enhances the inhibition of Frondoside A (Fr A) on UM-UC-3 cell viability and migration. Cells were treated with Frondoside A, CpG-ODN, and Frondoside A combined with CpG-ODN for 24, 48, 72 h. The effects of combinations of Frondoside A and CpG-ODN were tested in parallel with the drugs used alone (**A**). Effects of drugs combination with Frondoside A, and the combinational index (CI) was calculated with the CompuSyn v.1.0. software (**B**). Frondoside A significantly suppressed the migration of UM-UC-3 cells, cell mobility was measured at 0, 12 and 24 h (objective × 4) (**C**), and columns are means; bars are S.E.M. * Significantly different at *p* < 0.05. *** Significantly different at *p* < 0.001. n.s (not significant). “a” stands for versus blank control; “b” stands for versus EPI (10 μM); “c” stands for versus Frondoside A (0.5 μM) alone.

It can be seen in Figure 4A that, in comparison with individual Frondoside A treatment or CpG-ODN treatment, the combined Frondoside A and CpG-ODN treatment significantly reduced the cell growth at 24, 48, and 72 h. When used in combination, CpG-ODN significantly enhanced the inhibition of cell viability mediated by Frondoside A, indicating that CpG-ODN plays a potentiating role in inhibiting the cell viability. To further investigate the potential synergistic effects of Frondoside A in combination with CpG-ODN, the Chou-Talalay method was used. Based on the Chou-Talalay method, CI values lower than 0.85 or close to 1.2 indicate synergy or additive effects, respectively. Remarkably, Figure 4B shows that Frondoside A was strongly synergistic and additive when used in combination with CpG-ODN. Therefore, the combined concentrations at 0.5 μM of Frondoside A and CpG-ODN (0.001–1 μM) were selected for subsequent experiments to investigate the synergistic effects.

**Figure 5:**
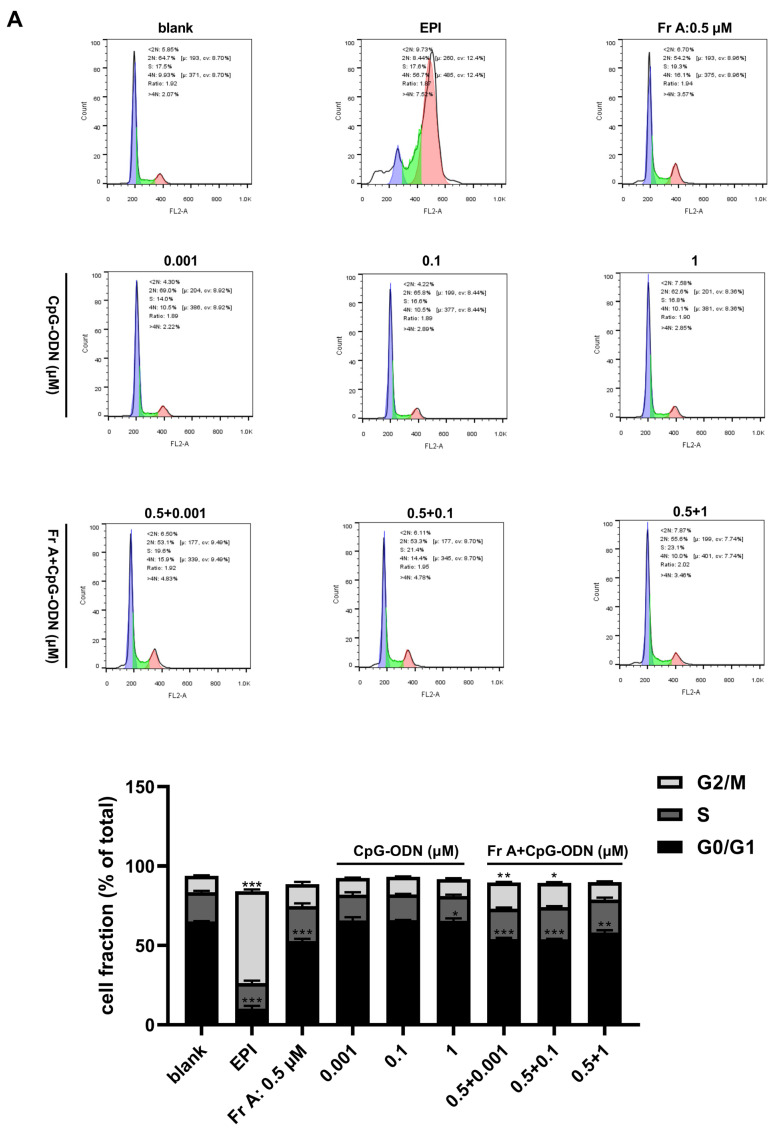

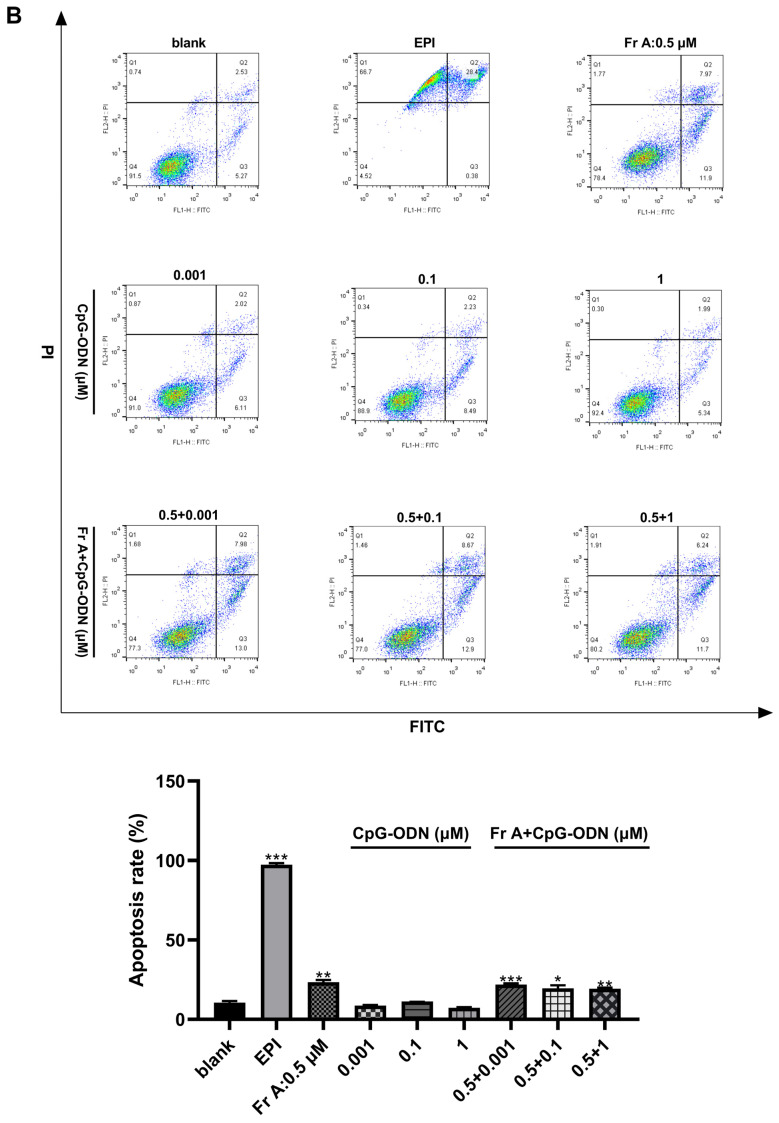

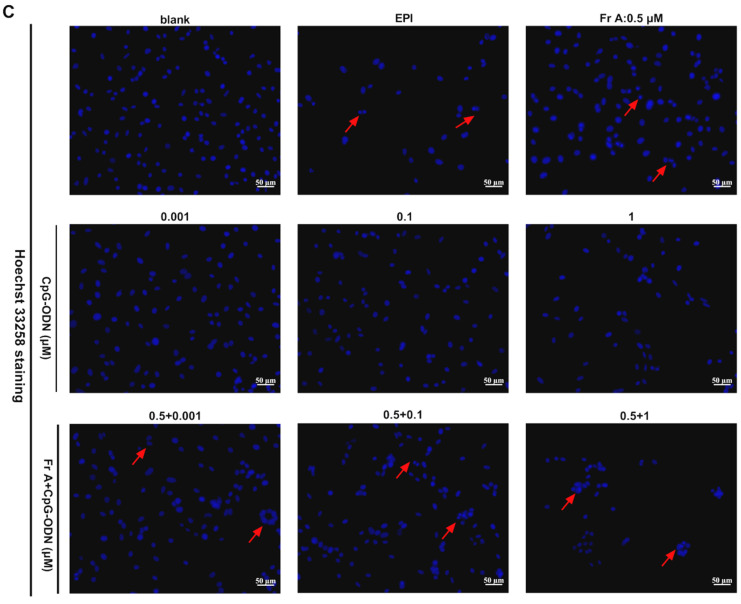
Effects of Frondoside A (Fr A) and CpG-ODN on cell cycle, apoptosis and nuclei morphology in bladder cancer cells. Cells were treated with Frondoside A (0.5 μM) and different concentrations of CpG-ODN (0.001, 0.1, and 1 μM) individually and in combination for 48 h. The distribution of cell cycle (**A**). The induction of cell apoptosis (**B**) and the morphological changes (The pictures were made at ×200) (**C**). Data represent the mean ± S.E.M. * Significantly different at *p* < 0.05. ** Significantly different at *p* < 0.01. *** Significantly different at *p* < 0.001.

The results of cell migration (i.e., cell scratch experiment) showed that the drug combinations of Frondoside A (0.5 μM) and CpG-ODN (0.001–1 μM) also resulted in more pronounced inhibition of cell migration than both EPI (10 μM, positive control) and Frondoside A (0.5 μM) alone (Figure 4C) at both 12 h and 24 h time points. The effects of the combinations of Frondoside A and CpG-ODN on cell cycle distribution and induce cell apoptosis were also studied. The combinations did not induce significant alteration in the cell cycle and cell apoptosis; as shown in Figure 5, it is clear that while combinations of Frondoside A (0.5 μM) and CpG-ODN (0.001–1 μM) had no significant effects on cell cycle and caused no detectable increase in the level of apoptosis in comparison with Frondoside A (0.5 μM) treatment alone, the combinations did significantly decrease the G0/G1 phase and increased G2/M phase (Figure 5A) and induced cell apoptosis and morphological changes in the UM-UC-3 cells (Figure 5B,C). The combination induced apoptosis rate was estimated to be 20.29% ± 0.81% (Figure 5B).

### 3.5. Frondoside A, CpG-ODN or in Combination Regulates the Expression of Target Genes in TP53 Signaling and Intrinsic Pathway

**Figure 6:**
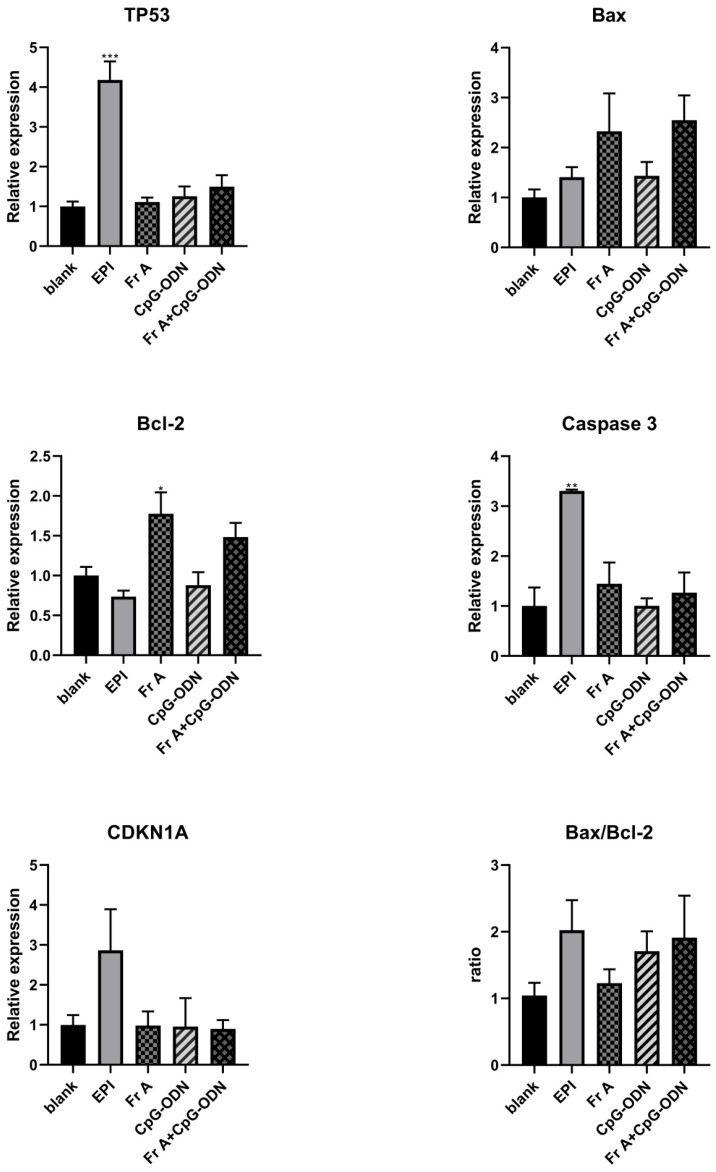
Effects of Frondoside A (Fr A), CpG-ODN individually and in combination in bladder cancer cells of TP53 signaling and Intrinsic pathways. Cells were treated with the indicated concentrations of Frondoside A and CpG-ODN for 48 h, EPI (10 μM) as positive control. Equal amounts of cell extracts were analyzed by Quantitative Real-Time Polymerase Chain Reaction assay with TP53, Bax, Bcl-2, Caspase 3, and CDK1A. Data represent the mean ± S.E.M. * Significantly different at *p* < 0.05. ** Significantly different at *p* < 0.01. *** Significantly different at *p* < 0.001.

It is evident from Figure 6 that, in comparison with EPI (10 μM), cells treated with either Frondoside A (0.5 μM), CpG-ODN (1 μM) or in combinations had different effects on the related gene expressions. The qPCR results revealed that the cells treated with Frondoside A (0.5 μM), CpG-ODN (1 μM) or in combinations for 48 h showed elevated levels of Bax and caspase 3 (markers of apoptosis) expressions and higher ratio of Bax/Bcl-2; however, none showed statistical significance. In addition, the level of the cyclin-dependent kinase inhibitor 1A (CDK1A) was unaltered in the UM-UC-3 cells for all treatments with the exception of the EPI treatment at 10 μM (Figure 6). After EPI treatment (10 μM), the expressions of TP53 and caspase 3 were significantly up-regulated in UM-UC-3 cells, while the Frondoside A (0.5 μM) significantly increased the Bcl-2 expression (Figure 6). 

### 3.6. The Suppression of Tumor Growth In Vivo

**Figure 7:**
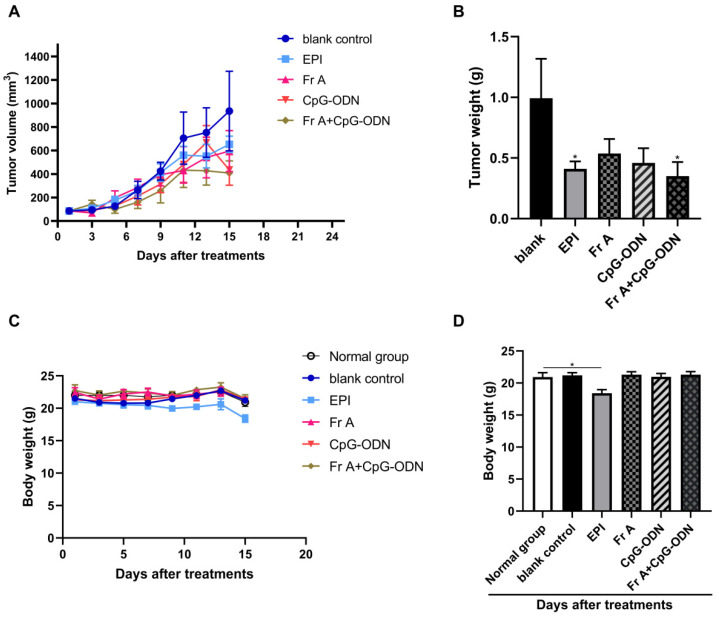
The suppression on the tumor growth of human bladder cancer xenografts. Bladder cancer cells UM-UC-3 (3 million per mouse) were subcutaneously transplanted into immune-deficient nude mice to establish a xenograft tumor model. Mice were treated with different drugs in 14 days. Tumor volumes were measured every 2 days to assess the effect of treatments on tumor growth (**A**). Xenografts were resected, and the weights of the tumors were measured at the end of the observation period (**B**). The body weight of the mice was monitored every 2 days to observe the drugs toxicity, and the body weight was weighed prior to the death (**C**,**D**). Data represent the mean ± S.E.M. * Significantly different at *p* < 0.05.

After treatments with Frondoside A (800 μg/kg/day), CpG-ODN (1 mg/kg/dose), EPI (1 mg/kg/dose) and the combination (Frondoside A and CpG-ODN), the growth of subcutaneously transplanted tumors were inhibited when compared with the blank control (0.9% normal saline). Similar differences were also found in tumor weight at the end of the experiment, both EPI group and the drug combinations of Frondoside A (800 μg/kg/day) and CpG-ODN (1 mg/kg/dose) were more effective than either individual drug alone (Figure 7A,B). It was evident that the body weight of animals throughout the entire course of the experiments showed a clear downward trend in the EPI group, whereas there was no obvious trend in the other treatment groups (Figure 7C). In fact, the body weight of mice in the group treated with EPI (1 mg/kg/dose) was significantly reduced (*p* < 0.05) compared with other groups, suggesting that treatments with Frondoside A and CpG-ODN at the indicated doses did not cause significant side effects in the tested mice (Figure 7D). It was also noted that there was no significant difference in organ coefficients between the experimental groups and the blank control group (data not shown). 

**Figure 8:**
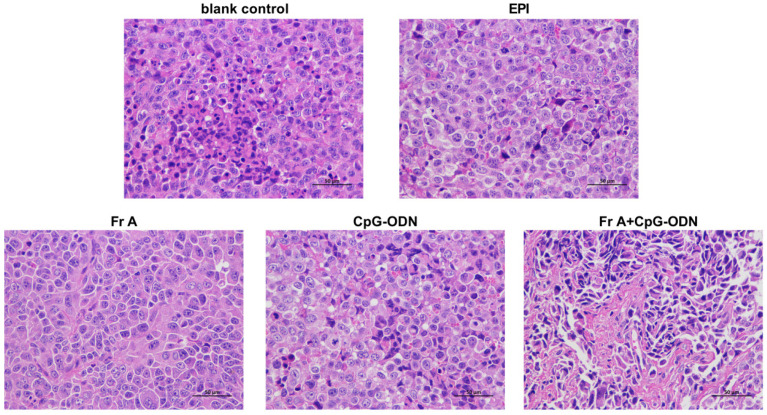
Histopathologic sections of tumor tissues (H&E, ×200). Nude mice were treated with different drugs in 14 days. All of the animals were sacrificed after the treatments and the tumors were collected for histological analysis.

As shown in Figure 8, cancer cells in all treatment groups exhibited relatively regular, sparse arrangement and wide cell space in comparison with blank control group (0.9% normal saline) (Figure 8), indicating that all the drug treatments have significant inhibitory effect on tumor growth. Interestingly, a closer look at the cell morphology indicates that the combination treatment group (i.e., Fr A+ CpG-ODN) showed distinctly different cell morphologies from other condition treatment conditions, suggesting that further in-depth investigation into the mechanism for the combination treatment is warranted in future studies (Figure 8).

## 4. Discussion

Bladder cancer is a highly prevalent disease worldwide. Chemotherapy is one of the most important treatment options for bladder cancer [3,35], particularly for late-stage bladder cancer with metastasis. Recently, significant progress has been made with new therapeutic options including chemotherapy, targeted therapy, and immunotherapy approved for bladder cancer treatment. However, drug-induced toxicities and drug resistance limit the efficacy and clinical applications of these drugs. Therefore, it is crucial to find novel anti-bladder cancer drugs from natural sources, especially marine organisms.

Sea cucumber (*Cucumaria frondosa*, *Holothuroidea*), as a highly nutritious medicinal and edible food, has both strong biological activity and excellent safety [6,8]. In particular, sea cucumber triterpenoid glycoside Frondoside A is the most researched group of compounds due to its potential anti-cancer activities [36]. It has been well established that Frondoside A treatments can specifically target cancer cells, such as pancreatic cancer, lung cancer, colon cancer, and prostate cancer cell lines while have minimal toxic effect on normal cells [13], suggesting that Frondoside A—a marine organism extract—possesses a strong anti-cancer activity with less side effects. Hence, in this study, we first investigated the anti-bladder cancer effects of Frondoside A alone or in combination with CpG-ODN on bladder cancer cell viability, apoptosis, cell cycle and migration in vitro, and subsequently their anti-bladder cancer effects in vivo. In the present study, Frondoside A alone or in combination with CpG-ODN decreased the viabilities of UM-UC-3 cells in a concentration-dependent manner; When used in combination with both Frondoside A (0.75 μM) and concentrations of CpG-ODN (0.001–1 μM), the combination of both drugs showed more inhibitory effects on cell growth than EPI (10 μM) at 24, 48, and 72 h. Therefore, Frondoside A showed a strong synergistic or additive effect when combined with CpG-ODN in UM-UC-3 cell cultures. This effect can be explained, at least partially, by the ability of Frondoside A to play a leading role in inducing cell apoptosis. It was shown that Frondoside A isolated from *Cucumaria frondosa* enhances the anti-proliferative effect of gemcitabine in pancreatic cancer, demonstrating additive effects when used in combination with gemcitabine [11]. Samir Attoub et al. found that combinations of Frondoside A with oxaliplatin or 5-Fluorouracil were significantly more effective in inhibiting colon cancer cell HT-29 proliferation and triggering apoptosis than using either of the cytotoxic agents alone [37]. Moreover, previous studies have also shown that Frondoside A can potentiate the anti-cancer effect of the conventional therapeutic agents in acute leukemia [21]. Thus, it can be concluded that Frondoside A from sea cucumbers can be used as a promising candidate to enhance the efficacy of anti-cancer drugs.

Apoptosis is one of the key mechanisms of anti-cancer drugs in cancer therapy and has emerged as an effective target for the discovery and development of novel anti-cancer agents [38,39]. Generally, the machinery of apoptosis is complex and involves many signaling pathways, including extrinsic or intrinsic apoptosis pathways. Previous studies showed that the sea cucumber triterpene glycoside Frondoside A induces the apoptosis through regulation of several pro-apoptotic factors, such as caspase-3, -8, and -9, PARP, Bax, p21, and inhibits autophagy in urothelial carcinoma cells [40]. Serget A. Dyshlovoy et al. demonstrated that Frondoside A induces non-classical apoptosis in human Burkitt lymphoma cells in a caspase-independent manner [41]. Although the underlying mechanism of cell apoptosis induction in cancer cells differs from others reported, it is interesting to note that the induction of apoptosis appears to be a common effector response in Frondoside A to cause tumor cell death. We found that Frondoside A induced cell apoptosis and cell cycle arrest in a concentration-dependent manner and showed morphological changes in apoptotic UM-UC-3 cells, such as the characteristic cytoplasmic cell shrinkage, budding of plasma membrane, and chromatin condensation.

Interestingly, we demonstrated that Frondoside A (0.5 μM) induced apoptosis did not alter TP53 and caspase-3 expressions, but up-regulated Bcl-2 gene, whereas when used together with CpG-ODN (1 μM) the drug combination showed elevated levels of Bax, Bcl-2, and ratio of Bax/Bcl-2 (data none showed statistical). In comparison, treatment with EPI (10 μM) could significantly activate the pro-apoptotic functions of TP53 and caspase-3 (*p* < 0.05). Therefore, our results suggest that Frondoside A and EPI use different apoptosis signaling pathways and different mechanisms in inducing apoptosis in bladder cancer cells. EPI may primarily affect apoptosis-related pathways, while triterpene glycosides Frondoside A affect apoptosis-related or invasion- and metastasis-related pathways. These mechanisms may explain the caspase-independent and TP53-independent character of Frondoside A induced apoptosis in bladder cancer cells. Although several target genes (TP53, Bax, Bcl-2, Caspase 3, and CDK1A) for these induction of apoptosis effects have been identified, which not be potential therapeutic targets of Frondoside A, the precise mechanisms underlying their effects are unknown. We speculate that Frondoside A possibly has other targets that are related to the apoptosis signal pathway. Therefore, the molecular mechanisms of Frondoside A and CpG-ODN may be involved in more complicated cell signaling pathways and molecular networks, which are needed to be further explored to reveal these underlying apoptosis mechanisms in depth.

As tumor metastasis and recurrence is the primary cause of cancer-related mortality among cancer patients, exploring drugs that can inhibit tumor migration is an effective strategy for bladder tumor therapy [42]. In this study, we provided the first evidence that Frondoside A does not only suppress proliferation of the UM-UC-3 cells but also exerts a strong inhibitory effect on the migratory property of the cancer cells. In addition, the anti-metastatic activity of Frondoside A was also significantly enhanced when combined with CpG-ODN. It is possible that Frondoside A can counter cell invasion by inhibiting cell migration and preventing bladder cancer cells becoming metastatic. Although we have demonstrated that Frondoside A has a significant effect on inhibiting bladder cancer cell migration, the specific molecular mechanisms remain to be identified in the future.

To observe the anti-bladder cancer effect of Frondoside A alone or in combination with CpG-ODN on tumor growth in vivo, UM-UC-3 xenografts were grafted in nude mice. We demonstrated slower tumor growth in all drug treatment groups when compared with the blank control treatment group (0.9% normal saline). Combination of Frondoside A and CpG-ODN treatments resulted in a remarkable potentiation (*p* < 0.05) of the Frondoside A therapeutic effects, while at the same time minimized the side effects of the treatment. The cancer inhibitory effects of Frondoside A have been observed previously in LNM35 lung, PC-3, DU145 prostate, and MDA-MB-231 breast xenografts in immune-deficient mice. Based on these results, we believe that our data further confirm that Frondoside A in combination with CpG-ODN has the potential to enhance the treatment of bladder cancers.

## 5. Conclusions

The sea cucumber triterpene glycoside Frondoside A showed potential anti-bladder cancer activity by inhibiting cell viability and migration, inducing the apoptosis and affecting cell cycle. CpG-ODN significantly improves the efficacy of Frondoside A in anti-bladder cancer in vitro and in vivo. These interesting results suggest that combined therapy of CpG-ODN with Frondoside A can be a promising approach in treating bladder cancer. This therapeutic strategy should be further considered as a candidate for neoadjuvant, adjuvant, and palliative treatment for bladder cancer patients.

## Figures and Tables

**Table 1:** Gene-specific primers used in the study.

Genes	Sequence (5′→3′)	
	Forward Primers	Reverse Primers
TP53	CCAGGGCAGCTACGGTTTC	CTCCGTCATGTGCTGTGACTG
Bax	CTTTTGCTTCAGGGTTTCATCCA	TCCATGTTACTGTCCAGTTCGT
Bcl-2 [28]	CTTCGCCGAGATGTCCAGCCA	CGCTCTCCACACACATGACCC
Caspase 3	CCAAAGATCATACATGGAAGCG	CTGAATGTTTCCCTGAGGTTTG
CDKN1A	TGTCCGTCAGAACCCATGC	AAAGTCGAAGTTCCATCGCTC
GADPH	TGACATCAAGAAGGTGGTGAAGCAG	GTGTCGCTGTTGAAGTCAGAGGAG

## Appendix A

The relevant structural information of Frondoside A can be searched and browsed in the PubChem (Molecular Formula: C_60_H_96_O_29_SNa; CAS number:127367-76-4; web site: https://chem.nlm.nih.gov/chemidplus/sid/0127367764, 30 November 2022).
